# Supplementary material for: Genetic Basis Identification of a NLR Gene, TaRPM1-2D, That Confers Powdery Mildew Resistance in Wheat Cultivar ‘Brock’
Source: Plants (Basel). 2025 Aug 26;14(17):2652. doi: 10.3390/plants14172652 (PMC12430440; doi:10.3390/plants14172652)
Supplement: Supplementary file 1 [file plants-14-02652-s001.zip › Supplenmental lengends-revised.pdf]

Table S1: Primer sequence in this study;

Table S2: Genetic analysis of resistance to *Bgt E09* in Jing411 /Brock;

Table S3: The annotated information of genes in the delimited physical interval;

Table S4: Statistics calculations of VIGS and OE;

Figure S1: *Pm2* sequence in Jin411 and Brock;

Figure S2: Comparison of predicted TaRPM1-2D amino acid sequence in Jin411 and Brock;

Figure S3: Analysis of *TaRPM1-2D* silencing efficiency. GKP Buffer: Brock infected with GKP Buffer, BSMV:*GFP*: Brock infected with BSMV:*GFP*, BSMV:*TaRPM1-2D-V*: Brock infected with BSMV:*TaRPM1-2D*;

Figure S4: Identification of transgenic wheat. A, genomic PCR identification of *TaRPM1-2D*. M:DL 5000 Marker；line 1: positive controls；line 2: negative controls；line 3-6: pTCK303-*TaRPM1-2D* transgenic wheat. B, relative expression levels of *TaRPM1-2D* in over-expression wheat plants.
