# Supplementary material for: Genetic Basis Identification of a NLR Gene, TaRPM1-2D, That Confers Powdery Mildew Resistance in Wheat Cultivar ‘Brock’
Source: Plants (Basel). 2025 Aug 26;14(17):2652. doi: 10.3390/plants14172652 (PMC12430440; doi:10.3390/plants14172652)
Supplement: Supplementary file 1 [file plants-14-02652-s001.zip › Table S1.pdf]

Table S1

Primer sequence in this study

Primer name	Sequence（5’→3’）	Usage
Pm2-F	ATGAATTAGCAAGCAATGTGTCCACC	Amplication of *Pm2*
Pm2-R	CTAGTGCAACGACGACTCGGAC	
TaRPM1-like-F	ATGCCGGAGATTGTGATTCTTCTAGC	Amplication of full length
TaRPM1-like-R	TCAATCCCGGAGAGTATACCGCC	
qPCR-RPM1-N-F	TTTGGGTGGACGACTGGC	Expression level analysis
qPCR-RPM1-N-F	CTCCGTTGTATGCTCTGGTG	
TaRPM1-like-OE-F	TCTAGAGGATCCCCGGGTACCATGCCGGAG ATTGTGATTCTTCTAGC	Construction of over-expression vector
TaRPM1-like-OE-R	TTCGAGCTCTCTAGAACTAGTTCAATCCCG GAGAGTATACCGCC	
TaRPM1-like-V1-F	GGGGTGAAAGCACATTAGAAGAA	Construction of VIGS vector
TaRPM1-like-V_1-R	CGAAGTCGGCGTACACTAGAAAA	
Ubi-F	CGGTCGTTCATTCGTTCTA	Identification of OE plants
TaRPM1-like-R（Ubi）	TACTTCCTCCAACCAGCCCTC	
Tactin-qRT-F	TACTCCCTCACAACAACCG	Internal gene of qRT-PCR
Tactin-qRT-R	AGAACCTCCACTGAGAACAA	
