# Supplementary material for: Genetic Basis Identification of a NLR Gene, TaRPM1-2D, That Confers Powdery Mildew Resistance in Wheat Cultivar ‘Brock’
Source: Plants (Basel). 2025 Aug 26;14(17):2652. doi: 10.3390/plants14172652 (PMC12430440; doi:10.3390/plants14172652)
Supplement: Supplementary file 1 [file plants-14-02652-s001.zip › Table S2.pdf]

Table S2 Genetic analysis of resistance to *Bgt E09* in Jing411 /Brock

Parent and hybrid progeny	Passing on from generation generation	Individuals or family coefficient	Plants observed			Expected ratio	χ^2	*P*
			HR	Seg	HS			
Jing411	P_S	30			30			
Brock	P_R	30	30					
$P_R \times P_S$	F_1	36	36					
	F_2	250	186		64	3:1	0.048	0.8
	$F_{2:3}$	659	173	321	165	1:2:1	0.632	0.7
