# Supplementary figures and images for: Genetic Basis Identification of a NLR Gene, TaRPM1-2D, That Confers Powdery Mildew Resistance in Wheat Cultivar ‘Brock’
Source: Plants (Basel). 2025 Aug 26;14(17):2652. doi: 10.3390/plants14172652 (PMC12430440; doi:10.3390/plants14172652)

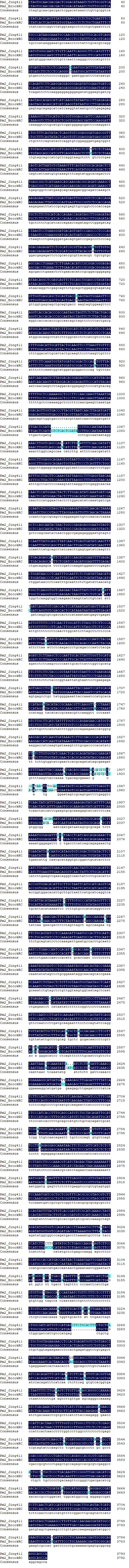

Supplement: Supplementary file 1 [file plants-14-02652-s001.zip › Fig. S1.jpg]

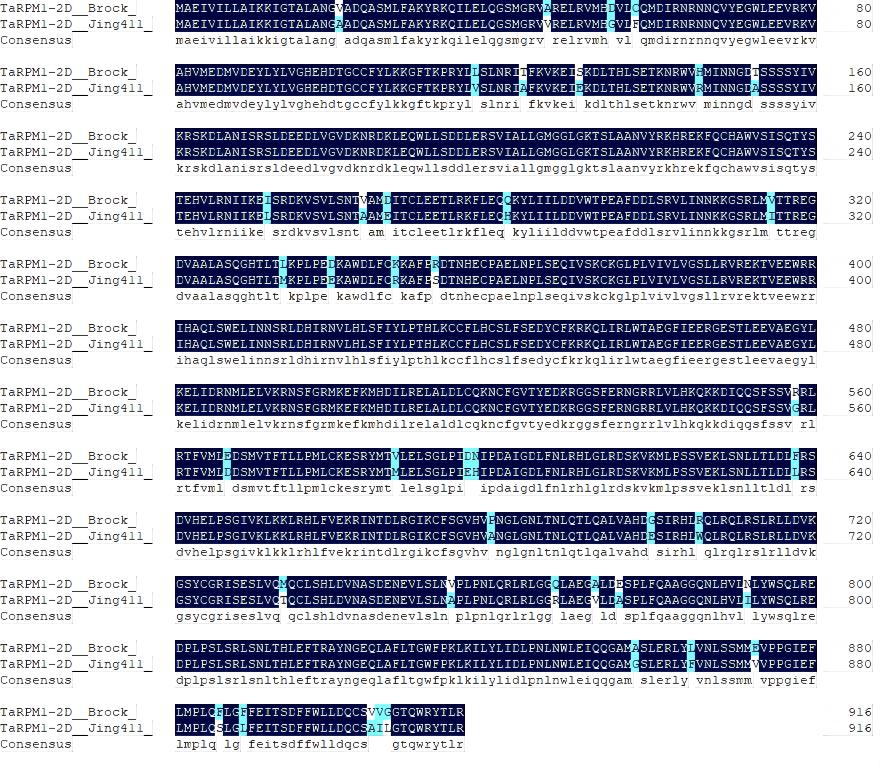

Supplement: Supplementary file 1 [file plants-14-02652-s001.zip › Fig. S2.jpg]

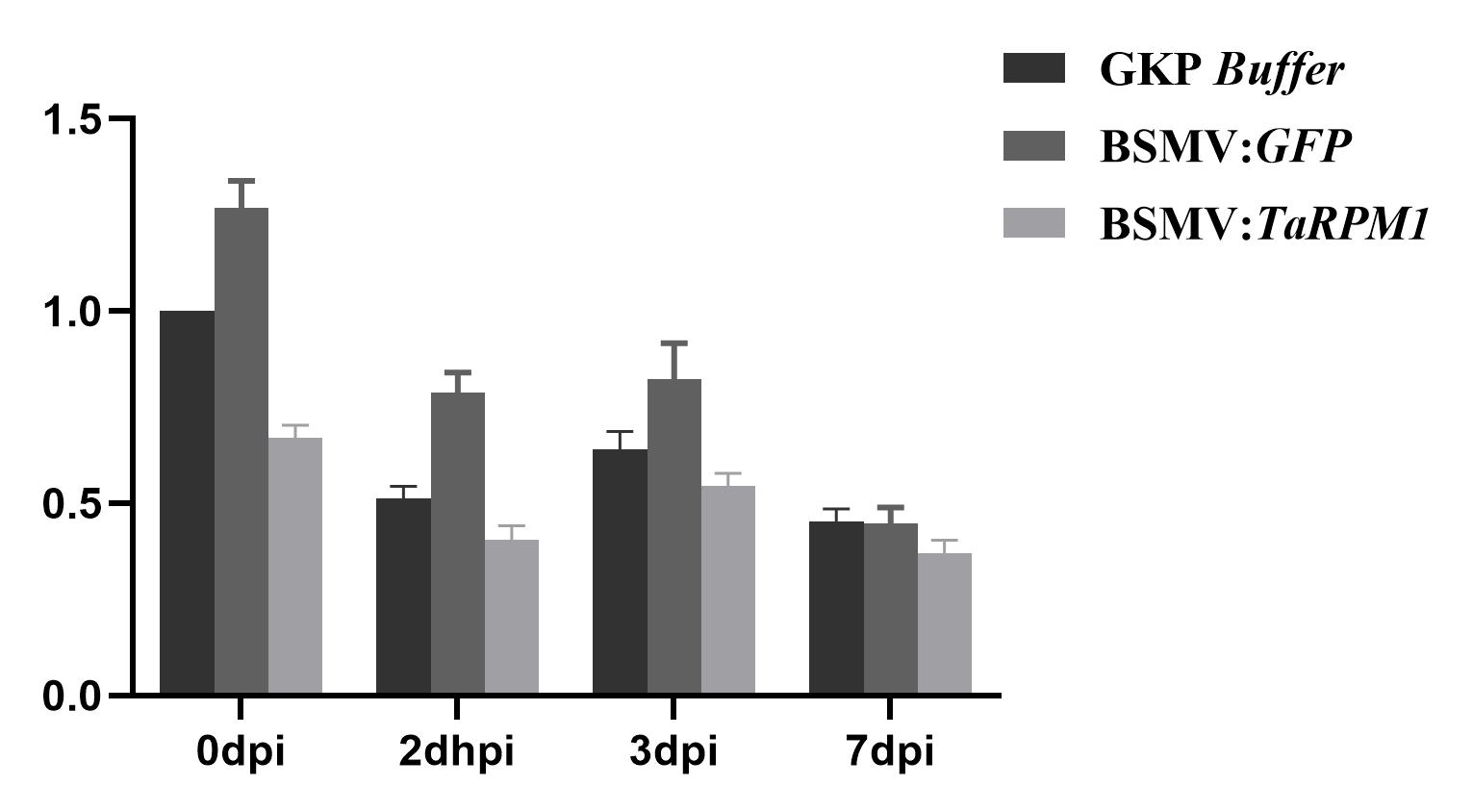

Supplement: Supplementary file 1 [file plants-14-02652-s001.zip › Fig. S3.jpg]

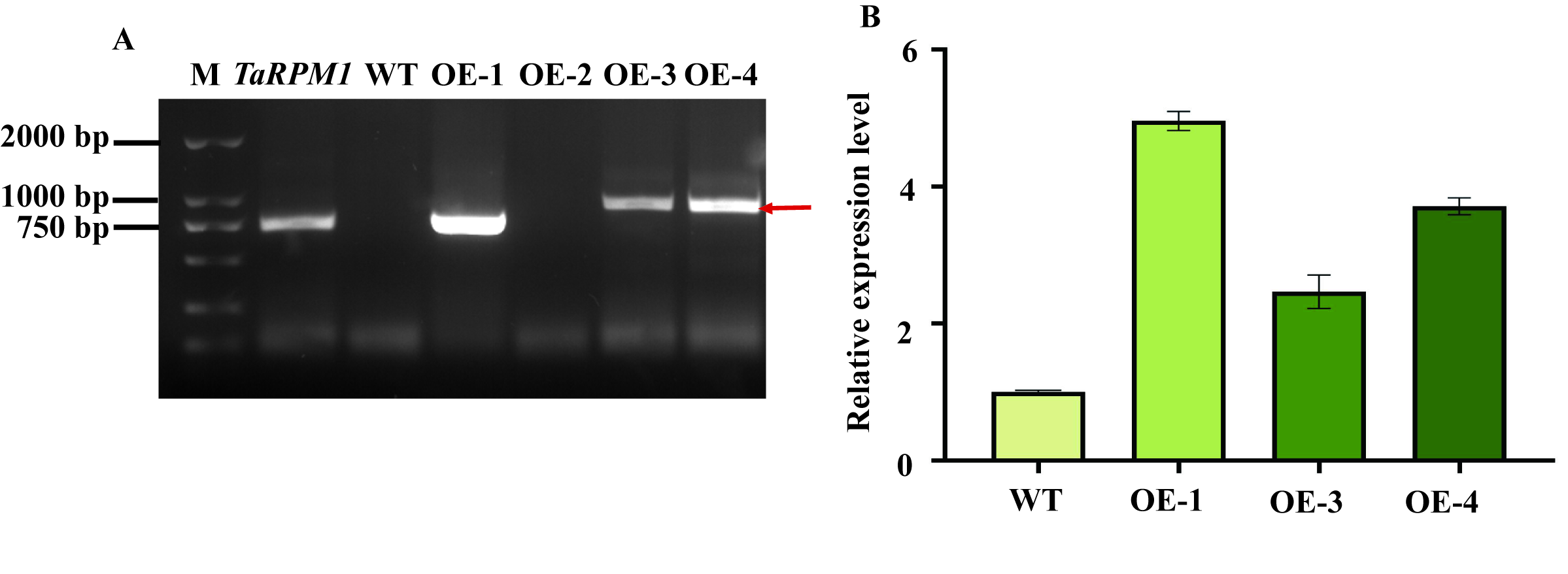

Supplement: Supplementary file 1 [file plants-14-02652-s001.zip › Fig. S4.tif]
